# Cardiac Telerehabilitation After Acute Coronary Syndrome Ensures Similar Improvement in Exercise Capacity as Inpatient Rehabilitation, Regardless of the Age Profile of the Compared Groups

**DOI:** 10.3390/jcm14093143

**Published:** 2025-05-01

**Authors:** Barbara Bralewska, Julia Wykrota, Małgorzata Kurpesa, Jarosław D. Kasprzak, Urszula Cieślik-Guerra, Ewa Wądołowska, Tomasz Rechciński

**Affiliations:** 1 Student Scientific Association, Medical University of Lodz, 90-419 Lodz, Poland; barbara.bralewska@student.umed.lodz.pl (B.B.); julia.wykrota@student.umed.lodz.pl (J.W.); 2 1st Department of Cardiology, Medical University of Lodz, 90-419 Lodz, Poland; malgorzata.kurpesa@umed.lodz.pl (M.K.); jaroslaw.kasprzak@umed.lodz.pl (J.D.K.); 3 Department of Cardiac Rehabilitation, The Bieganski Hospital, 91-347 Lodz, Poland; cieursz@gmail.com (U.C.-G.);

**Keywords:** cardiac rehabilitation, COVID-19, telerehabilitation

## Abstract

**Introduction:** During the COVID-19 pandemic, the availability of cardiac rehabilitation (CR) was limited. On the other hand, during that period of epidemic restrictions, patients with acute coronary syndrome (ACS) required careful control and monitoring after coronary events. The aim of this study was to assess whether CR conducted during the epidemic restrictions in a remote mode ensured similar improvement in physical performance as CR conducted in a centre-based mode before the COVID-19 pandemic. **Material and Methods**: In this one-centre study, we compared the demographic and clinical profiles of patients after ACS who completed inpatient CR before the COVID-19 era with those of patients who completed telerehabilitation during the COVID-19 pandemic. We assessed the workload on the initial and final exercise tests (ExT) obtained by patients and compared the values of the differences between the final and initial ExT. The study included 359 patients (pts) participating in inpatient CR before October 2020 (the suspension of centre-based CR) and 60 pts who took part in telerehabilitation after July 2021 (the introduction of the tele-CR programme). Both inpatient and tele-CR were performed according to the guidelines of the Working Group for Cardiac Rehabilitation of the National Cardiac Society. A telemedic platform was used to control ECG, blood pressure and body mass of the pts participating in telerehabilitation. **Results**: The improvement of physical performance did not differ significantly between the two groups. The pts who completed telerehabilitation were significantly older than those who completed inpatient CR. The values of other parameters, such as the percentage of females, BMI, the percentage of pts with arterial hypertension and type 2 diabetes mellitus, as well as left ventricular ejection fraction did not differ significantly between the compared groups. Nor did the results of initial ExT expressed in METs, the results of final ExT and the improvement of workload understood as the difference between the final and initial results of ExT differ significantly—they were 7.7 ± 3.06 vs. 7.89 ± 2.98 with *p* = 0.82; 9.08 ± 0.29 vs. 8.98 ± 3.48 with *p* = 0.84, and 1 [0–2.2] vs. 1.2 [0–1.8] with *p* = 0.17, respectively. **Conclusions**: In our centre, telerehabilitation after acute coronary syndrome guaranteed an equally good improvement in physical capacity as that observed in inpatient CR patients, regardless of the difference in the age profile of the compared groups. These results encourage the popularization of telerehabilitation with remote monitoring of ECG, blood pressure and body mass.

## 1. Introduction

According to the guidelines of the European Society of Cardiology (ESC), comprehensive cardiac rehabilitation (CR) is classified as class IA as a method of treating patients after myocardial infarction, after myocardial revascularization, with chronic coronary syndrome, and with heart failure. In Poland, until 12 December 2015, telemedicine functioned rather as a theoretical concept, without any real possibility of its application. It was not until the Act of the 9th of October 2015 on the change of the Act on the Healthcare Information System and of certain other Acts (Journal of Laws, item 1991) and the amendment of the Act on the Medical and Dental Professions in December 2015 that its practical application was possible. One of the factors which had a major impact on the rapid development of cardiac telerehabilitation was the COVID-19 pandemic, when the availability of inpatient cardiac rehabilitation (CR) was completely abolished, and patients immediately after acute coronary syndromes (ACS) required careful monitoring and provision of rehabilitation opportunities.

The aim of this study was to assess whether remote cardiac rehabilitation conducted during the epidemic period (the study group) provided a similar improvement in exercise capacity as inpatient rehabilitation before the COVID-19 pandemic (the reference group).

## 2. Materials and Methods

In this retrospective study, we analysed data from 60 patients in the study group, who participated in cardiac telerehabilitation between 30 July 2021 and 30 June 2022. The total number of patients who participated in telerehabilitation was 98; those who were rehabilitated for reasons other than acute coronary syndrome, e.g., after valve replacement, electrotherapy, congestive heart failure, or Bentall surgery, were excluded. The final number of patients who participated in the study was 60. Among the women in the study group, one (9%) was professionally active, while the remaining 10 (91%) were retired. Among the men, 15 (31%) were professionally active and 34 (69%) were retired. In the reference group were 359 patients who participated in inpatient rehabilitation in the years preceding the pandemic at a tertiary referral centre. The patients in the study group and in the reference group were referred for rehabilitation after ACS and percutaneous coronary angioplasty or coronary artery bypass grafting.

In accordance with the requirements of the insurer (the National Health Fund), the telerehabilitation programme [1] took place during 24 sessions within a maximum of 90 consecutive days. Our centre preferred the patients to have rehabilitation sessions without breaks other than days off from work, but if necessary, the patient could take breaks between sessions. On the first day of the programme, the patient was admitted to the centre and qualified for the rehabilitation programme by a cardiologist. This qualification consisted of conducting an interview with the patient; a physical examination; determining their pharmacological treatment and its compliance with ESC standards (the percentages of patients from the study group taking various medications are presented in Table 1); analysis of the laboratory tests performed so far; measuring the patient’s blood pressure, heart rate, body mass and height; and also an assessment of the patient’s mental state by a psychologist. Patients were also checked for any contraindications to rehabilitation, such as anaemia with haemoglobin concentration below 10 g/l, inflammation, unstable angina pectoris, myocarditis, pulmonary embolism, peripheral embolism, heart failure in the period of decompensation or atrioventricular conduction disorders requiring the implantation of a cardiac pacemaker.

On the second day of the programme, the physician qualifying the patient for telerehabilitation performed an electrocardiographic stress test (ECG) according to the Bruce-Ramp protocol, assessed their heart rhythm, heart rate limit, and checked for the presence of electrocardiographic signs of myocardial ischemia or new disturbances of rhythm or conduction.

**Figure 1:**
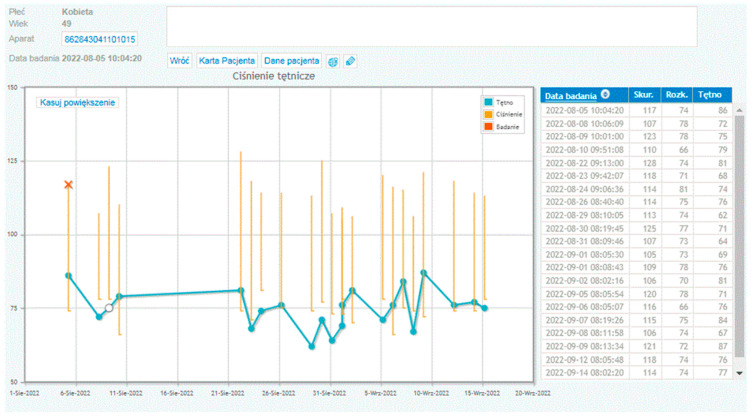
An example of a graph showing the blood pressure and the heart rate of a patient participating in telerehabilitation.

**Figure 2:**
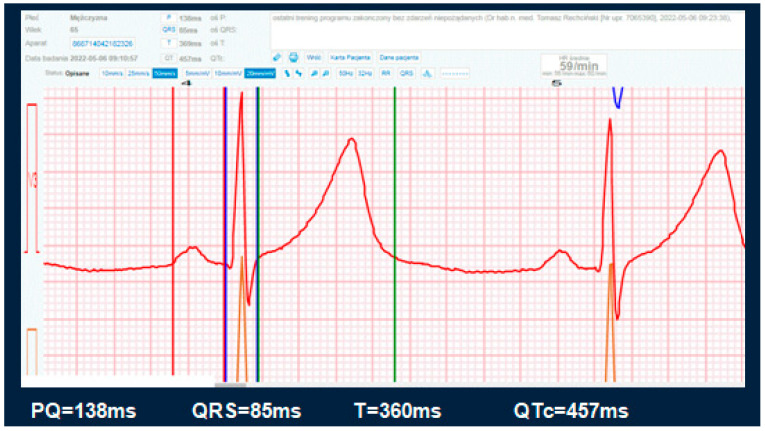
An example of measurement of the duration of the PQ interval (between red and purple lines), the QT interval (between purple and green lines) and the QRS complex (between purple and blue line).

On days 3, 4, and 5 of the programme, patients from the study group were educated on how to use the telerehabilitation device. This included learning how to use the telerehabilitation monitoring equipment: measuring heart rate, blood pressure, body mass, following the planned exercise programme, and assessing their level of exertion according to the Borg scale. The patients also took part in meetings with a psychologist, who helped them to cope with possible symptoms of depression and anxiety related to the disease, and with a dietitian, who taught them the principles of preparing a healthy diet. On days 6–23, the actual rehabilitation of the patients took place. The training protocol was standardised for all patients. The training consisted of marching under heart rate control (the patients were advised to reach a training heart rate limit). Before each training session, during a telephone conversation, the patients answered questions about their current health, well-being, and medications taken. Then, they sent their resting ECG record as well as their blood pressure and body mass measurements to the monitoring centre—the doctor also had all previous measurements in the form of a graph (see Figure 1). The resting ECG was sent to the centre twice before the start of training, and then at 20 min intervals and after the training. In the transmitted ECG, it was possible to assess heart rhythm: whether it was sinus rhythm or not, whether there were arrhythmias (e.g., atrial fibrillation, premature ventricular or supraventricular extrasystoles). The system also allowed the measurement of the duration of the PQ interval, of the QT interval and of the QRS complex (Figure 2). If there were no contraindications, the patient received consent to start the training session. The telerehabilitation ECG was monitored several times during each session. For stationary patients, ECG was performed during the first three days of the programme and then only in the case of emerging complaints (palpitations, retrosternal pain). In order to improve exercise capacity, three forms of exercise were recommended: endurance training, respiratory muscle training, and resistance and stretching training. On the last day, a final exercise ECG test was performed according to the Bruce–Ramp protocol.

Having been educated in the use of the telerehabilitation device, the patients performed training outside the centre; this included walking, cycling, and training on a stationary cycloergometer or treadmill (if available). If the training went according to plan, immediately after the training session, the telerehabilitation device, following a signal from the patient, sent an ECG to the monitoring centre via a mobile phone network. All the data sent by the patient were collected, analysed and stored in the monitoring centre in order to assess the safety, effectiveness and correctness of the rehabilitation carried out by the patients. On the basis of the assessment of the intensity of the exertion as perceived by the patients according to the Borg scale and of the electrocardiograms recorded during the training, decisions were made about the further rehabilitation process, and especially about the possibility or necessity of increasing or decreasing the load.

For patients from the reference group, training at the inpatient centre included morning gymnastics, endurance training on a cycloergometer (twice a day for 10–20 min), resistance training on a multi-gym, a rowing machine or an elliptical bike, Nordic walking and breathing training. The patients had also specially organized meetings with a psychologist, who conducted relaxation therapy, and with a dietitian, who analysed individual eating habits and recommended dietary modifications if necessary.

Both cardiac telerehabilitation and inpatient rehabilitation were conducted in accordance with the guidelines of the Working Group on Cardiac Rehabilitation of the Polish Cardiac Society. The electrocardiogram, blood pressure and body mass of patients participating in the remote rehabilitation program were monitored by means of the telemedicine platform manufactured by Pro-PLUS S.A. Polska.

Statistical analysis included the Mann–Whitney U test, Student’s *t*-test and chi-square test.

## 3. Results

Patients from both groups were compared in terms of demographic and anthropomorphic as well as clinical characteristics, and exercise test results. The results are presented in Table 2 and Table 3.

Statistical analysis showed a statistically significant difference only in the age of patients participating in the study in comparison with the reference group. The median age for the group of centre-based patients was 60.15, and for the patients participating in telerehabilitation it was 63.7.

There was also a numerical difference between the percentage of women in the study group and in the reference group, but (like the difference in mean BMI values) it turned out to be statistically not significant. Pearson’s linear correlation coefficient between age and the final outcome of the exercise test was r = −0.048 with a determination coefficient of R^2^ = 0.002, whereas the correlation between BMI and the final outcome of the exercise test was r = −0.329 and R^2^ = 0.108. Weight control was mainly aimed at assessing fluid retention in patients taking diuretics. In the group monitored during the pandemic, we did not observe the need to change the dose of diuretics. None of the patients participating in the telerehabilitation were taking loop diuretics.

**Figure 3:**
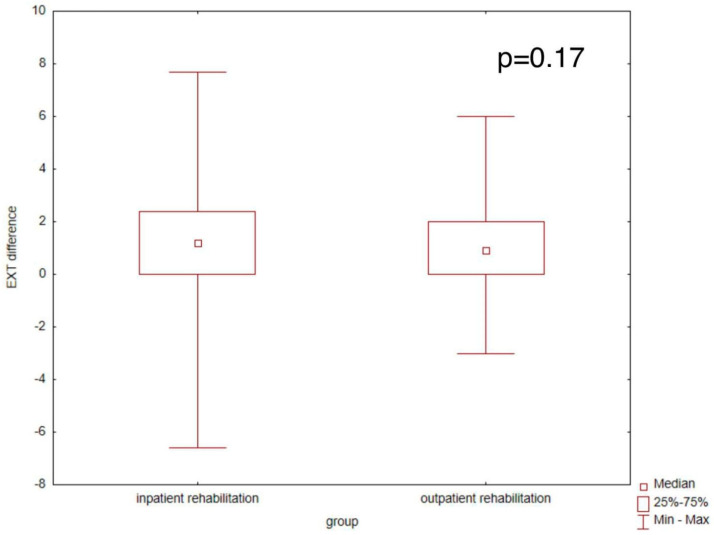
A comparison of patients from the inpatient rehabilitation group and patients participating in telerehabilitation in terms of increased exercise capacity. Y−the difference between the exercise capacity in the final exercise test and the result in the initial exercise test.

The result of the comparison of the increase in exercise capacity expressed in METs in the final exercise test as compared to the initial test and a comparison of these differences between the groups of patients from the centre-based group and those participating in telerehabilitation indicate no significant differences, as presented in Figure 3.

During the telerehabilitation of patients from the study group, adverse events occurred, presented in Table 4. However, they did not ultimately affect the deterioration of the final results of rehabilitation in terms of exercise capacity. They did not require interruption of the rehabilitation process and they disappeared when the pharmacotherapy used was modified or when new drugs were introduced [2].

## 4. Discussion

The most important result of our work has been to show that there is no statistically significant difference in the improvement in exercise capacity between the group of patients participating in cardiac telerehabilitation and the group of patients participating in inpatient rehabilitation at the centre as assessed by an electrocardiographic exercise test performed according to the Bruce–Ramp protocol. This study also answers the question often asked by patients before they start cardiac telerehabilitation, which is this: what will be the probable result of the improvement in exercise capacity after the completion of the rehabilitation programme, and will it be similar to what they would obtain by participating in inpatient cardiac rehabilitation?

A statistically significant difference in age was found between the study group and the reference group. To determine if it could have any impact on the results, further research in this area would be needed. Another limitation of our study is the lack of information about digital literacy of patients participating in telerehabilitation.

Studies conducted to date indicate that during cardiac rehabilitation, patients improve their exercise capacity by an average of 1.5 METs [3]. Uznańska-Loch et al. [4] showed in their study that the highest probability of achieving the maximum improvement in exercise tolerance after cardiac rehabilitation is associated with the co-occurrence of left ventricular ejection fraction of >43% and a result of ≤8.4 METs in the initial exercise test. Age, BMI or sex were not significantly associated with the degree of improvement in exercise tolerance. Significant differences in the results occur in patients with normal and with reduced ejection fractions [5]. Heart failure with reduced left ventricular ejection fraction is a strong predictor of mortality after ACS on admission. Differences in the results achieved during cardiac rehabilitation are also related to gender [6]. Women achieve less improvement during cardiac rehabilitation, which can be explained by the higher age of the female population with ACS and with worse clinical results upon admission to the rehabilitation programme. The conclusion from the study by Bielecka-Dąbrowa et al. (the aim of which was to implement a health care model for patients with heart failure (HF) and to assess clinical and psychosocial differences between women and men in the study population) provides information that women suffer from HF symptoms more often and have a worse quality of life as assessed by the EQ-5D-5L than men, despite a better left ventricular ejection fraction [7].

Most studies conducted by other centres provide results similar to ours—cardiac telerehabilitation brings comparable results to centre-based cardiac rehabilitation in terms of exercise capacity and quality of life.

The study by Piotrowicz et al. [8], conducted, however, before the pandemic, included 152 patients with heart failure, mean age 58.1 ± 10.2 years, NYHA class II and III, and left ventricular ejection fraction ≤40%. The patients were randomly assigned to either home-based cardiac rehabilitation with ECG telemonitoring (n = 77) or conventional outpatient cardiac rehabilitation (n = 75). All the participants completed an eight-week cardiac rehabilitation programme. The two groups were comparable in terms of demographic and clinical characteristics, as well as pharmacological treatment. The efficacy of cardiac rehabilitation was evaluated on the basis of changes in the New York Heart Association (NYHA) functional class, in peak oxygen uptake (VO_2_ peak) during cardiopulmonary exercise testing, in the distance achieved in the 6-min walk test, and in the scores from the SF-36 quality of life questionnaire. Significant improvements were observed in all the parameters assessed in both the standard rehabilitation group and the telerehabilitation group.

Further evidence supporting the efficacy of telerehabilitation is provided by the study conducted by Avila et al. [9], in which 90 patients with coronary artery disease (including 80 men) were randomly allocated in a 1:1:1 ratio to three groups: home-based rehabilitation (n = 30), centre-based rehabilitation (n = 30), and a control group receiving no rehabilitation (n = 30). The findings demonstrated that home-based exercise rehabilitation was as effective as the traditional, supervised centre-based programme. Importantly, no significant differences were observed between these two intervention groups in terms of final exercise capacity or physical activity levels.

In a separate study, Bernocchi et al. [10] investigated the feasibility and effectiveness of a four-month integrated home-based telerehabilitation programme (Telereab-HBP) in patients diagnosed with chronic obstructive pulmonary disease (COPD) and coexisting chronic heart failure (CHF). The primary outcome measure was the distance covered in the six-minute walk test (6MWT), while secondary outcomes included time to clinical events (hospitalization and mortality), the severity of dyspnoea, exercise activity levels, functional disability, and health-related quality of life. The Telereab-HBP intervention comprised a remote monitoring of cardiorespiratory parameters, weekly nurse-led telephone consultations, and a structured exercise regimen supervised weekly by a physiotherapist. Outcomes were reassessed following a two-month, non-interventional follow-up period. A total of 112 patients were randomised evenly between the intervention and the control groups (n = 56 per group), with a mean age ± standard deviation of 70 ± 9 years; 82.1% of participants were male. Interestingly, a comparable gender distribution was observed in our study, with 82% of participants in the telerehabilitation group and 72% in the inpatient rehabilitation group being male. The findings reported by Bernocchi et al. demonstrated a significant improvement in the 6MWT distance following the four-month programme, leading to the conclusion that this approach—combining remote physiological monitoring and regular professional consultations—is both feasible and efficacious in elderly patients with concurrent COPD and CHF.

In the study by Kraal et al. [11], 90 patients at a low or moderate cardiovascular risk, who were starting cardiac rehabilitation, were randomised to 3 months of home-based training with telemonitoring advice or to in-centre training. Physical fitness improved significantly at discharge and at one-year follow-up (*p* < 0.01) in both groups, with no significant differences between the two groups. The levels of exercise activity did not change significantly during the one-year follow-up. The health care costs were numerically lower in the “home” group, but this difference was not statistically significant. The authors of that study reached similar conclusions to the ones that we present in our article: telerehabilitation yielded equally good results as in-hospital cardiac rehabilitation. Although adherence to the training recommendations was similar in both groups, satisfaction with the rehabilitation programme was higher in the home-based group. Although in our study we did not conduct a survey on the degree of satisfaction with the form of rehabilitation, no patient reported any problems with operating the telerehabilitation equipment or barriers related to this form of training.

In the randomised study conducted by Bravo-Escobar et al. [12], 28 patients with stable coronary artery disease and moderate cardiovascular risk were enrolled. Fourteen patients were assigned to a conventional hospital-based cardiac rehabilitation program (the control group), while the remaining 14 participated in a home-based program with mixed supervision (the intervention group). The average BMI and mean age of the participants were comparable to those observed in our study. Notably, as in our cohort, the home-based group had a slightly higher mean age than the hospital-based group. No significant differences were observed between the two groups regarding exercise duration, METs achieved during the stress test, or the 1-min recovery index—all of which improved in both groups following the rehabilitation programme.

An interesting modern method for monitoring the course of telerehabilitation is the use of smartwatches by patients described by Mitropoulos et al. [13]. A randomised trial was designed to assess the effects of real-time monitoring of cardiac telerehabilitation using smartwatches in people who had recently had a myocardial infarction. All the patients completed baseline and follow-up measurements after 24 weeks, which assessed peak oxygen uptake, the mean daily step count, distance, and calories. The online group showed similar improvements in the mean daily step count and the mean daily walking distance after 24 weeks as the gym-based exercise group. There were no adverse events related to exercise during the study. The results of the study indicate that a real-time supervised exercise programme of cardiac rehabilitation using smartwatch technology to monitor the haemodynamic response in patients with coronary artery disease and after a myocardial infarction is as effective as the gym-based exercise programme.

Researchers also compared the effectiveness of outpatient hospital-based cardiac rehabilitation and telerehabilitation in patients after coronary artery bypass grafting (CABG) surgery [14]. Similarly to our study, the mean age of patients in the “home group” was slightly higher than the mean age of patients in the “hospital group”. Measurements encompassed incremental shuttle walk test (ISWT) performance, psychosocial questionnaire scores, and body composition, measured at baseline, at the end of the eight-week intervention, and again at the end of a four-week post-intervention follow-up. Both the home-based and hospital-based cardiac rehabilitation groups demonstrated improvements in ISWT duration compared to baseline values. Home-based rehabilitation proved to be equally effective as traditional outpatient rehabilitation in enhancing exercise capacity among post-coronary artery bypass grafting patients. Furthermore, the home-based approach appeared to be superior in sustaining these gains over the follow-up period.

Interesting results are presented in the study by Gu et al. [15]. They analysed a cohort of stable patients with coronary artery disease (CAD) who had undergone percutaneous coronary angioplasty and who participated in two different modes of the cardiac rehabilitation programme after being discharged from hospital. CR took place in two periods: from January 2019 to December 2019 (inpatient CR) and from May 2020 to May 2021 (remote CR). Exercise capacity was assessed using the 6 min walk test (6MWT), oxygen uptake at peak exercise (VO2max), and anaerobic respiratory threshold (VO2AT) before discharge, after 8 weeks of CR and after 12 weeks of CR, performed either in the centre or remotely at home. No adverse events occurred during the programme. Patients from both groups increased their walking distance in the 6MWT test with higher VO2max after 8 and 12 weeks. The patients participating in telerehabilitation showed better results in terms of anxiety and depression levels in comparison with the patients from inpatient rehabilitation.

Other cardiology studies on telemedicine raise important and interesting issues. The aim of a prospective study of Mariani et al. [16] was to analyse the feasibility and effectiveness of virtual visits compared to traditional in-person visits and to evaluate the experience of patients receiving remote care in clinical electrophysiology. The study focused on the following indicators: (1) improvement in complaints after the initial visit, (2) resolution of remote monitoring (RM) alerts during the follow-up, (3) need for emergency hospitalisation and (4) patient satisfaction as assessed using the Patient Satisfaction Questionnaire-18 (PSQ-18). Compared with in-person visits, virtual visits produced comparable results in terms of reduction of the number of RM alerts and of the frequency of symptoms at follow-up, with no differences in terms of urgent hospitalisation. Furthermore, patients rated virtual visits as more satisfactory than in-person visits. These results confirm that remote care is not only feasible, but also as effective as traditional care, with high levels of patient satisfaction.

The aim of the study of Ankışhan [17] was to develop new speech signal features to capture the relationship between voice recordings and blood pressure (BP). In this context, the literature describes a database containing the vowels/a/, recorded at different blood pressure values, under controlled environmental and room conditions. Tools such as convolutional neural networks for regression (CNN-R), support vector machines for regression (SVM-R) and multilinear regression (MLR) were used to predict BP values on the basis of extracted features. The experiments showed that the highest accuracy in predicting systolic BP values was achieved using CNN-R for the vowels/a/. The results obtained suggest that the proposed feature vector (FVx) reflects the relationship between blood pressure and vocal traits, opening up the possibility of using it in patient BP monitoring systems.

The limitation of our work is the small size of the study group, but this group comes from a unique period during the COVID-19 pandemic, when the only form of cardiac rehabilitation for patients after a myocardial infarction was telerehabilitation. Gathering a similar group of patients in the current period—outside the period of restrictions related to the pandemic—would be impossible owing to the fact that currently, patients and doctors have a choice between three forms of cardiac rehabilitation—inpatient rehabilitation, outpatient rehabilitation in the centre, and telerehabilitation (outside the centre).

## 5. Conclusions

In our centre, telerehabilitation after acute coronary syndrome performed during the COVID-19 pandemic guaranteed an equally good improvement in exercise capacity as that observed in patients undergoing inpatient rehabilitation, regardless of the difference in the age profile of the compared groups. These results, compared with studies from different centres and from different periods, encourage the popularization of the mode of cardiac rehabilitation based on the remote monitoring of ECG, arterial blood pressure and body mass.

## Figures and Tables

**Table 1:** Percentage comparison of the medications taken by patients in the study group.

Class of Medications	The Percentage (%) of Patients Taking the Medications
Statins	97
Beta blockers	94
Angiotensin-converting enzyme II inhibitors	90
Acetylsalicylic acid	85
Proton pump inhibitor (pantoprazole)	77
P2Y12 inhibitors	74
Calcium channel antagonists	22
Aldosterone receptor antagonists	18.3
Ezetimibe	18.3
Flozins	18.3
Trimetazidine	15
Thiazide diuretics	15
Ivabradine	10
Sartans	8.3

**Table 2:** A comparison of the demographic, anthropomorphic, and clinical characteristics of patients.

Demographic and Anthropomorphic Characteristics of Patients				
Variable	The Study Group (Telerehabilitation)	The Reference Group (Inpatient CR)	Significance	All Rehabilitation Patients Participating in the Study
Number of patients	60	359	-	419
Age [years]	63.7 ± 10.8	60.15 ± 9.8	0.003	60.66 ± 10
Females; number [%]	11, [18.3]	99, [27.5]	0.64	114, [27.2]
BMI	29.41 ± 4.65	28.0 ± 4.5	0.14	28.55 ± 4.5
Clinical characteristics				
Arterial hypertension; number [%]	40, [66.6]	255, [71.03]	0.49	295, [70.41]
Diabetes mellitus, number [%]	15, [25.0]	84, [23.39]	0.79	99, [23.63]
Left ventricular ejection fraction [%] mean ± SD	46.95 ± 8.16	47.48 ± 9.03	0.47	47.41 ± 8.91

**Table 3:** A comparison of the results of exercise tests of the patients participating in the study.

A Comparison of Physical Performance Between the Two Groups				
Variable	The Study Group (Telerehabilitation)	The Reference Group (Inpatient CR)	Significance	All Rehabilitation Patients Participating in the Study
Initial exercise test [MET]	7.89 ± 2.98	7.72 ± 3.06	0.82	7.75 ± 3.1
Final exercise test [MET]	8.98 ± 3.48	9.03 ± 3.29	0.84	9.03 ± 3.3
Improvement in workload capacity [MET] median [25–75%]	1.2 [0–1.8]	1.0 [0–2.2]	0.17	1.0 [0–2.2]

**Table 4:** The percentage of adverse events in patients from the study group.

Adverse Events in Patients from the Study Group	The Percentage of Occurrence (%)
Suboptimal results of arterial blood pressure measurements	16.7
Bradycardia	6.7
A worsening of heart failure symptoms	5.1
Resting heart rate >75/min	3.3
Urinary tract infections	1.7
Episodes of atrial fibrillation	1.7

## Data Availability

The source data available from the corresponding author.
